# End‐Of‐Life Care in a Cardiac Intensive Care Unit: A Retrospective Analysis of Interventions During the Last 48 Hours

**DOI:** 10.1111/nicc.70717

**Published:** 2026-09-30

**Authors:** Eduardo Tavares Gomes, Larissa Braga Celes, Geovana Aparecida dos Reis Cirino

**Affiliations:** ^1^ Federal University of Minas Gerais Belo Horizonte Minas Gerais Brazil

**Keywords:** cardiology, critical care, critical care nurses, intensive care units, palliative care

## Abstract

**Background:**

Palliative care in intensive care units should be planned jointly with patients and their families and documented in medical records. Proximity to death should prompt reassessment of the therapeutic strategy.

**Aim:**

To analyse the interventions performed during the last 48 h in patients who died in the cardiac intensive care unit.

**Study Design:**

A retrospective cohort study was conducted in the cardiac intensive care unit of a large university hospital in Southeastern Brazil between March and April 2025. The deaths that occurred in 2022, 2023 and 2024 were evaluated.

**Results:**

A total of 83 deaths with more than 5 days of hospital admission were analysed. There were few records documenting patients' and families' wishes. Only 12 (14.5%) records of limitations of life‐sustaining treatments and 11 (13.3%) records of do‐not‐resuscitate orders directed to the healthcare team were identified.

**Conclusion:**

Interventions intended to modify the course of the disease exceeded those aimed at palliative care in our cohort.

**Relevance to Clinical Practice:**

These findings may support the implementation of trigger‐based protocols for palliative care assessment in cardiac ICUs, the development of training strategies focused on communication and documentation of patient preferences for ICU teams, the strengthening of the role of the multidisciplinary team, and standardized comfort‐focused interventions during the last 48 h of life.

## Impact Statements

- What is known about the topic
  ○ Palliative care in intensive care units should be planned jointly with patients and their families and documented in medical records.
  ○ Proximity to death should prompt reassessment of the therapeutic strategy.
- What this paper adds
  ○ In this context, interventions intended to modify the course of the disease exceeded those aimed at palliative care alone.
  ○ Patients presented complications and such a severe critical condition prior to the last 48 h of life might have no significant deterioration that could clearly indicate the onset of terminality.
  ○ Perceiving the final 48 h and the dying process probably becomes difficult when patients remain critically ill from admission or for a prolonged period before death.

## 1 Introduction

End‐of‐life care has become a central component of contemporary cardiac intensive care units (CICUs). Population ageing, multimorbidity and prolonged survival with complex or end‐stage cardiovascular disease have transformed the profile of critically ill cardiac patients, who frequently experience high symptom burden and psychological distress despite access to advanced life‐sustaining technologies such as mechanical ventilation, mechanical circulatory support and renal replacement therapies [1, 2, 3]. In this context, many CICU admissions represent the final stage of life, and decisions about when to transition from aggressive disease‐directed interventions to comfort‐focused care are increasingly frequent and ethically challenging [1, 4, 5].

Recent data from cardiac and general ICUs show that death often follows explicit decisions to limit or withdraw life‐sustaining treatments rather than treatment failure alone [4, 5, 6, 7]. In a large contemporary North American CICU registry, most patients who died had a prior decision for comfort measures only; however, these decisions tended to occur several days after admission, and in the majority of cases, death followed within hours of this change in goals of care.4 Similar patterns of late or infrequent use of do‐not‐resuscitate orders, therapeutic ceilings and device deactivation, as well as low systematic involvement of palliative care teams, have been described in Italian CICU networks and single‐centre coronary intensive care cohorts [1, 3, 8]. These findings suggest a persistent tendency towards intensive use of invasive therapies in the last days of life, with limited formalization of end‐of‐life decisions.

At the same time, there is a growing consensus that early identification of patients at high risk of death, proactive serious illness communication and structured approaches to withholding or withdrawing life‐sustaining therapy can reduce non‐beneficial interventions and improve the quality of dying for patients and families [5, 7, 9, 10]. International guidelines and umbrella reviews on ICU end‐of‐life care emphasize timely integration of palliative care, standardized assessment and treatment of pain, dyspnoea, and agitation and family centred decision‐making as key domains of high‐quality care [5, 7, 9, 10, 11]. In CICUs specifically, palliative care involvement and goal‐of‐care discussions have been associated with higher rates of redirection towards comfort care and withdrawal of disproportionate treatments, without prolonging length of stay or time to death [2, 4, 12].

In this scenario, examining in detail the interventions performed during the final 48 h of life in a cardiac ICU provides a privileged window into the intensity and appropriateness of care at the end of life [6, 10]. By characterizing the use, escalation, or de‐escalation of advanced life support, the timing and documentation of treatment limitation decisions and the implementation of comfort‐oriented measures in this critical period, it becomes possible to identify potentially futile practices, gaps in palliative integration and opportunities to align care more closely with patient values and goals [4, 6, 9, 10]. Such analyses can inform targeted quality‐improvement strategies in cardiologic intensive care, including triggers for early palliative care consultation, protocols for withdrawal of life‐sustaining therapy, and educational interventions for multidisciplinary teams [3, 5, 11, 13].

A worldwide study on end‐of‐life care in the ICU showed that approximately 40% of patients who die in this setting have cardiovascular causes, with minor variations across regions of the world [6]. Discussions regarding the withdrawal and withholding of life‐sustaining interventions have been challenging for these patients [6]. In this study, the limitation of life‐sustaining efforts included ventilatory support (intubation and mechanical ventilation), cardiovascular support (use of vasopressors and resuscitation), renal support (renal replacement therapies) and nutritional support (enteral or parenteral) [6].

These issues represent a significant source of controversy among healthcare professionals caring for patients with severe and life‐threatening diseases. An inappropriate decision may lead to avoidable suffering, adding to the already tense context of end‐of‐life care in the ICU [7, 13]. In 2024, an important milestone for the field was the publication of a Brazilian document representing the position of the Bioethics Committee of the National Academy of Palliative Care (ANCP) [14]. This position, according to international guidelines [7], establishes principles to guide, from a bioethical perspective, the management of situations related to palliative care and the reflections required when choosing between maintaining disease‐modifying treatments or initiating palliative measures in ICUs [14].

In a context in which increasingly advanced life‐support technologies are implemented in critical care, and where clarity is essential when managing cases approaching death, it is of utmost importance to carefully consider and reconsider each decision. This study aimed to analyse the interventions performed during the last 48 h in patients who died in a cardiac intensive care unit. Given that euthanasia and assisted death are not permitted in Brazil, there were no patients in the study population who underwent a planned death.

## 2 Design and Methods

### 2.1 Setting and Sample

This retrospective cohort study was conducted in the cardiac intensive care unit of a large university hospital in Southeastern Brazil. The methodological steps followed the recommendations of the Strengthening the Reporting of Observational Studies in Epidemiology (STROBE) statement for cohort studies [15]. Data collection was conducted between March and April 2025.

The cardiological intensive care unit that served as the study setting had 18 beds exclusively dedicated to the care of patients with cardiac diseases, ranging from acute conditions, such as myocardial infarction and severe arrhythmias, to cardiac surgery, haemodynamic and electrophysiological procedures, and heart transplantation. This hospital has a palliative care consultation service that serves the ICUs, as well as an in‐house chaplaincy service. However, there are no protocols widely disseminated among ICU teams regarding the screening of palliative care needs and referral to the specialized service. In addition, families may visit any patient between 10:00 AM and 6:00 PM; however, when patients are elderly, family members may remain in the ICU 24 h a day, in compliance with Brazilian legislation.

The study population was defined based on the medical records of patients who died in the unit, including all cases that occurred between 2022, 2023 and 2024. Patients who died within less than 5 days of ICU admission and those who died due to non‐cardiac causes were excluded. Although there are accurate instruments to identify palliative care needs even in emergency situations, deaths occurring within the first 5 days of ICU stay were excluded because it was not possible, in such cases, to sensitively identify clinical progression between admission and the last 48 h of life [16]. In addition, another study in the cardiological ICU setting found that, when a consultation with a specialized palliative care service occurred, it took place from the fifth day of ICU stay onwards [17].

This sample is representative of end‐of‐life care in the ICU. Not all of these patients had consultations with the palliative care team or clearly defined strategies from that perspective, such as Do Not Resuscitate orders or other interventions. Patients undergoing planned death were not included in the population, given that euthanasia and assisted death practices are not permitted in Brazil.

### 2.2 Data Collection Tools and Methods

The cases were analysed using a structured form designed for the researchers' review of the electronic medical records. This form was developed using the Research Electronic Data Capture (REDCap) online platform. The first section of the instrument included case identification data, followed by information related to hospitalization, ICU clinical course and records of procedures and complications. Data were collected from the fifth day of admission and during the last 48 h before death, considering the entire period from hospital admission to death.

The procedures and records were primary outcomes and evaluated were categorized into those with a therapeutic intent—when terminality and palliative care were not discussed and the therapeutic plan was maintained with the same orientation and goals previously established—and those with a palliative intent—when there was documentation of deliberation to establish a new direction in the therapeutic plan owing to the proximity of death.

For data collection, the researchers received training on the scope of the study, the concepts used in the form, and the application of the instrument. Subsequently, an initial review of all deaths that occurred during the study period (*N* = 295) was conducted to classify the cases according to the inclusion and exclusion criteria. A total of 212 cases were excluded for not meeting the criteria because death occurred within less than 5 days of ICU admission (*N* = 108), patients were admitted for non‐cardiac causes (*N* = 42), or for both reasons (*N* = 62).

Following this stage, a pilot test was conducted to refine the instrument and align the assessments and criteria among the researchers. Ten cases were initially reviewed. After resolving doubts and making minor adjustments to the initially proposed form, the data collection phase was initiated.

### 2.3 Data Analysis

The data were entered into a database hosted on the REDCap platform, which enhanced data security and storage reliability. A significance level of 5% and 95% confidence interval were adopted for all statistical tests. The normality of variable distributions was assessed and confirmed using the Kolmogorov–Smirnov test, and parametric tests for comparison of proportions were applied (chi‐square and Fisher's exact tests). All analyses were performed using the IBM SPSS Statistics version 26. The significance level was set at *p* < 0.05.

### 2.4 Ethical and Institutional Approvals

This study was approved by the Research Ethics Committee of the institution (Approval No. 7.453.129, 20 Mars, 2025). Data were obtained from secondary sources (electronic medical records), and no patients or healthcare professionals responsible for the records were identified.

## 3 Results

A total of 83 deaths with more than 5 days of hospital admission were analysed. The cases included in the study had a mean age of 61.6 ± 13.8 years, ranging from 18 to 89 years of age. The mean length of stay between admission and death was 16.9 ± 10.9 days, with a range of 6–72 days. Fifty‐five (66.3%) patients were male and 28 (33.7%) were female. Overall, 54 (65.1%) patients were admitted for clinical reasons, such as acute coronary syndrome and arrhythmias, and 29 (34.9%) were admitted for surgical reasons (perioperative period).

The main comorbidities identified were arterial hypertension (42, 52.5%), type 2 diabetes mellitus (30, 37.5%) and smoking (11, 13.5%). Among the cases studied, 33 (41.2%) patients had a previous diagnosis of heart failure, and 22 (27.5%) had coronary artery disease. A large proportion (55%, 68.7%) had a previous diagnosis of or received a diagnosis of Chagas disease during hospitalization.

Table 1 presents the complications recorded during hospitalization, before and during the last 48 h of life of the patients included in the study (Table 1). Cardiogenic shock and arrhythmias requiring pharmacological reversal had the highest incidence at admission, and no complication showed a significantly higher incidence during the last 48 h of life (Table 1).

**TABLE 1:** Comparison of complications recorded during hospitalization before and during the last 48 h of life in the cardiac intensive care unit.

Complications during hospitalization (*N* = 83)	Before the last 48 h (*N*/%)	During the last 48 h (N/%)	p*
Non‐sinus bradycardia	8/9.6	6/7.2	0.576
Aborted cardiopulmonary arrest	17/20.5	20/24.1	0.574
Bleeding	9/10.8	3/3.6	0.072
Cardiogenic shock	57/68.7	14/16.9	< 0.01
Septic shock	24/28.9	31/37.3	0.528
Sepsis	22/26.5	15/18.1	0.191
Bloodstream infection	12/14.5	5/6.0	0.122
Ventilator‐associated infection	13/15.7	9/10.8	0.493
Other infections (urinary, wound, etc.)	17/20.5	11/13.3	0.301
Pressure injury	26/31.3	15/18.1	0.070
Stroke	6/7.2	1/1.2	0.117
New myocardial infarction	6/7.2	1/1.2	0.117
Acute respiratory failure	23/27.7	13/15.7	0.090
Non‐dialytic acute kidney injury	13/15.7	4/4.8	0.034
Dialytic acute kidney injury	32/38.6	31/37.3	0.980
Arrhythmias requiring electrical cardioversion	10/12.0	3/3.6	0.080
Arrhythmias reversed with pharmacological cardioversion	27/32.5	7/8.4	< 0.01

*Note:* Data collected in a cardiac intensive care unit, Belo Horizonte, Brazil, 2025. *p*‐value for the chi‐square or Fisher's exact test, as appropriate. It was possible to record more than one complication in some cases.

Regarding documentation in medical records of patients' and families' wishes related to end‐of‐life decisions and procedures, such records were infrequent in the present study. Among patients' wishes, there were (3.6%) three records of refusal of invasive procedures and two (2.4%) records documenting the wish not to be intubated, resuscitated, undergo surgery and return home (Table 2). Regarding the wishes of family members or legal representatives, three (3.6%) records documented requests not to intubate and resuscitate (Table 2). There were also records of consultation with the hospital palliative care team in eight (9.6%) cases, and only one (1.2%) case of religious visitation was requested by the patient or family.

**TABLE 2:** Recorded wishes of patients and family members/legal representatives regarding end‐of‐life care.

Patient's recorded wishes (*N* = 83)	*n*	%
Refusal of invasive procedures	3	3.6
Not to be intubated	2	2.4
Not to be resuscitated	2	2.4
Not to undergo surgery	2	2.4
To be discharged home	2	2.4
Not to receive blood products	1	1.2
To be transferred to the ward	1	1.2

Family/legal representative's recorded wishes	*n*	%
Not to intubate	3	3.6
Not to resuscitate	3	3.6
Not to be transferred to the ward	1	1.2
Not to receive blood products	1	1.2
Not to undergo surgery	1	1.2
Refusal of invasive procedures	1	1.2
Refusal of pharmacological treatment	1	1.2
To be discharged home	1	1.2

Records of procedures performed during the last 48 h with therapeutic intent were also reviewed. Among these, capillary blood glucose monitoring (48, 57.8%), initiation of sedoanalgesia (28, 33.7%), initiation of haemodialysis (30, 36.1%) and initiation of mechanical ventilation (26, 31.3%) were the most frequently documented procedures (Table 3).

**TABLE 3:** Therapeutic procedures performed in the last 48 h.

Therapeutic procedures in the last 48 h (*N* = 83)	*n*	%
Capillary blood glucose monitoring	48	57.8
Haemodialysis	30	36.1
Initiation of analgosedation	28	33.7
Initiation of mechanical ventilation	26	31.3
Initiation of antibiotics	23	27.7
Cardiopulmonary resuscitation	20	31.3
Central venous catheter insertion	15	18.1
Initiation of vasopressor drugs	14	16.9
Invasive arterial catheter insertion	11	13.3
Initiation of advanced support—intra‐aortic balloon pump	8	9.6
Urinary catheter insertion	7	8.4
Initiation of advanced support—epicardial pacemaker	4	4.8
Initiation of antiarrhythmic drugs	4	4.8
Regular insulin by continuous infusion pump	4	4.8
Initiation of advanced support—pulmonary artery catheter	3	3.6
Volume resuscitation (fluid replacement)	3	3.6
Electrical cardioversion	2	2.4
Nasoenteral tube insertion (for enteral feeding)	2	2.4
Non‐invasive ventilation	2	2.4
Initiation of advanced support – trans‐thoracic pacemaker	1	1.2

*Note:* It was possible to record more than one procedure in some cases.

Similarly, procedures performed during the last 48 h with palliative intent, aimed at promoting comfort and controlling or relieving symptoms, were examined. The total number of records with this intent was significantly lower. The main procedures in this category were the prescription of scheduled analgesia (19, 22.9%), documentation of the limitation of life‐sustaining treatments (12, 14.5%) and do‐not‐resuscitate orders directed to the healthcare team (11, 13.3%) (Table 4).

**TABLE 4:** Palliative procedures performed in the last 48 h.

Palliative procedures in the last 48 h (*N* = 83)	*n*	%
Analgesia at fixed times	19	22.9
Documentation of limitation of therapeutic efforts	12	14.5
Documentation of do‐not‐resuscitate order—guidance to the team	11	13.3
Cessation of antibiotic therapy	8	9.6
Cessation of enteral feeding	8	9.6
Documentation of do‐not‐resuscitate order after discussion with family	8	9.6
Removal of intra‐aortic balloon pump	7	8.4
Initiation of palliative sedation (including dexmedetomidine)	6	7.2
Administration of morphine by infusion pump	5	6.0
Administration of morphine at fixed times	4	4.8
Analgesia prescribed as needed – according to patient's complaint	3	3.6
Administration of morphine at the patient's request	2	2.4
Discontinuation of vasopressor drugs	2	2.4
Oral feeding with food from outside the hospital at the patient's request	2	2.4
Removal of indwelling urinary catheter	1	1.2
Reduction of sedation	1	1.2

*Note:* It was possible to record more than one complication in some cases.

There were no records of hypodermoclysis or withdrawal of invasive monitoring. Regarding multidisciplinary team care during the last 48 h of life, psychologists provided care to seven patients and five families and social workers assisted two families. There was only one record of psychiatric consultation and one visit by the chaplain.

Regarding pain assessment, 64 patients were evaluated using a numerical verbal rating scale and 42 were evaluated using the Behavioral Pain Scale. More than one option could be selected in this item, considering that during the last 48 h, there were changes in the level of consciousness and initiation of sedation, requiring changes in pain assessment strategies. There were five cases with no record of pain assessment during the last 48 h of life. These records were made by nursing technicians and nurses, with only one instance of pain assessment being documented by a physician. Among all pain‐related records, only seven cases documented the outcomes of interventions for pain management.

## 4 Discussion

The predominance of male patients and the high burden of comorbidities (hypertension, diabetes, smoking, heart failure and coronary artery disease) reflect patterns described in contemporary cardiac ICU registries [4, 18]. The high proportion of Chagas disease (nearly 70%) highlights a markedly different scenario from that observed in North American or European studies, where this aetiology is virtually absent [4, 5, 18]. This has important implications for prognosis, symptom burden (such as arrhythmias and cardiogenic shock) and palliative care planning.

The high incidence of cardiogenic shock and reversible arrhythmias at admission, without a significant increase in new complications during the last 48 h of life, suggests that many patients remain in advanced organ failure from an early stage, following a prolonged trajectory of decline. Multicentre cardiac ICU registries demonstrate a similar pattern, with intensive use of advanced life support (mechanical ventilation, circulatory support) preceding decisions focused on comfort measures [3, 18].

In the intensive care context, some palliative care interventions are delivered through consultative teams, while others should be provided by the primary ICU care team [5, 19]. The cardiac ICU team should, for example, ensure pain and symptom control, assess distress in patients, families and the healthcare team itself, discuss therapeutic plans and decision‐making and continuously reassess goals of care [5, 19]. Beyond this, a specialized palliative care team external to the ICU can contribute to the management of refractory pain and symptoms, spiritual, psychological and/or social support, mediation of decision‐making conflicts, discharge and transfer to wards, hospices or home care and bereavement support [5, 19].

A study conducted in a coronary ICU found that when a palliative care consultation was requested, it occurred only after the fifth day of hospitalization. Conversely, the authors reported that after myocardial infarction, palliative care measures and discussions focusing on comfort are generally considered only once complications arise, such as the need for nitroglycerin, mechanical support, stroke or other major complications [17].

An important study conducted in the United States found that between 2000 and 2017, the proportion of myocardial infarction cases receiving palliative care consultation in the cardiac ICU increased from less than 1% to 11.5% [20]. This group of patients was characterized by older age, a higher burden of complications, more frequent multiorgan failure and lower rates of percutaneous coronary intervention or surgical revascularization. These patients were also far more likely to have a documented do‐not‐resuscitate order [20].

During the last 48 h of life, procedures with therapeutic intent predominated (glycaemic control, initiation of sedoanalgesia, haemodialysis and mechanical ventilation). Explicit palliative interventions, such as scheduled analgesia—at fixed times, limitation of life‐sustaining treatments and do‐not‐resuscitate orders, were far less frequent. This pattern is consistent with reviews describing end‐of‐life management in ICUs as often aggressive, with late discontinuation of life support and limited emphasis on comfort and family support [8, 9, 12, 21, 22, 23].

Regarding the procedures performed, those classified as having therapeutic intent occurred without discussions about palliative care and were oriented towards cure and ICU discharge in the near future, whereas those classified as having palliative intent were preceded by discussions regarding measures to be taken from this perspective [14].

One particularly relevant finding merits reflection: no complications showed a significantly higher incidence during the last 48 h of life. This indicates the degree of clinical severity already present before this period, making it difficult to determine when critical illness transitions into an actively dying process.

In the Brazilian context, in line with international standards, the National Academy of Palliative Care (ANCP) defines treatments as futile in the strict sense when they have no possibility of achieving their intended physiological goal [14]. Procedures capable of prolonging life but with questionable ethical justification are considered to be potentially inappropriate [14]. The withdrawal or withholding of such potentially inappropriate life‐sustaining treatments requires explicit agreement on therapeutic goals, considering patients' values and perspectives, with formal documentation in the medical record [14]. These procedures entail social, emotional, and financial costs for families, healthcare teams and the healthcare system [14, 24, 25].

Globally, the limitation of life‐sustaining treatments has increased significantly [6, 26]. A study including 2056 patients admitted to surgical ICUs found that the main strategies for limiting life‐sustaining treatments involved withholding interventions (83.8%), occurring nearly six times more frequently than withdrawal of interventions already in place [27]. The most common measure identified was the do‐not‐resuscitate order [27]. Another large study conducted in Singapore showed a much smaller difference between withholding and withdrawing interventions, demonstrating that social and cultural factors are important predictors of these decisions [28].

Although it is neither intended nor appropriate to judge the merit of interventions based solely on the limited documentation in medical records, this analysis remains relevant. Therapeutic procedures performed during the last 48 h of life, such as initiation of haemodialysis or mechanical ventilation, were not accompanied by documented discussions with families or the healthcare team. Detached from discussions of palliative care and terminality, these procedures cannot be classified as inappropriate [14]. An important review on this topic suggests that when approaching families, clinicians should first explore patients' preferences as known by relatives, as well as their values and perspectives on life, and then present intervention options aligned with these preferences, clearly outlining the limits of medicine regarding prognostic certainty [29].

Multidisciplinary discussions about procedures promote professional engagement and improve not only involvement but also emotional and psychological adaptation to caring for patients at the end of their lives [24]. A recent study found that nursing professionals are more accepting of practices and interventions when they align with their own concepts of what constitutes a good death. This should further encourage the participation of all stakeholders in decision‐making, with the potential to minimize ethical conflicts and compassion fatigue [24]. Another study involving intensive care nurses showed that nursing procedures focused on person‐centred care are better accepted than complex, technology‐intensive interventions that may be considered unnecessary at the end of life (such as mechanical ventilation, ECMO and surgical interventions) [30].

There were few medical record entries documenting patients' and families' wishes regarding end‐of‐life decisions and procedures in the study. In large cardiac ICU registries, transitions to comfort‐focused care are documented in approximately two‐thirds of patients who die, although often late in the course of illness [3, 5]. International guidelines emphasize that documenting preferences and goals of care is central to avoiding futile treatments and aligning care with patient values [7, 9, 14].

Conversely, some procedures were identified as having been preceded by discussions about terminality. The main procedures in this category were the prescription of scheduled analgesia, documentation of the limitation of life‐sustaining treatments and do‐not‐resuscitate orders directed to the healthcare team. However, fewer than one‐quarter of deaths had records of this nature. This further reinforces the need to rethink the quality of palliative care in the ICU setting. Well‐established screening protocols to identify palliative care needs and integration with consultative services external to the ICU team might have resulted in more documentation and a greater number of patients experiencing a higher quality dying process.

Communication must be clear and avoid language that leads patients or families to believe that only one of the available options regarding the use or non‐use of life‐sustaining measures will relieve their suffering. Another publication in this field outlined five core principles for palliative care in the cardiac ICU: (1) clear, compassionate and timely communication among professionals; (2) decision‐making focused on patients' preferences, goals and values; (3) patient‐centred care to maintain comfort, dignity and personhood; (4) family centred care with open communication with the care team and access to the patient; and (5) bereavement care for families [5].

## 5 Limitations

The present study is limited in its ability to support broader inferences and generalizations, as the primary data source, the medical record, does not contain sometimes complete documentation, particularly regarding the subjective aspects involved in communication and supportive care. In addition, the small number of records documenting palliative care intentions makes it unclear to what extent the principles of this approach were actually applied in practice, but are underreported. This is a very important clinical question, but this paper is hypothesis‐generating at best. The data are very limited and cannot support broad generalizations about clinical care in cardiac ICUs in Brazil. Furthermore, this serves as a reminder of the importance of the professional document for every discussion—whether within the team or with the patient and family—regarding end‐of‐life decisions. Since this is a single ICU study, generalizability is limited; however, the research method—a retrospective review of the end‐of‐life experience of patients who died in the ICU from cardiac causes—may inspire other services to conduct similar studies and reflect on their own results.

## 6 Implications for Practice

The specific context of this study, characterized by a high prevalence of Chagas disease and a relatively younger patient population compared with studies from other countries, makes this cohort particularly relevant for low‐ and middle‐income countries, where the integration of palliative care into ICU practice remains incipient.24.

These findings may support the implementation of trigger‐based protocols for palliative care assessment in cardiac ICUs, the development of training strategies focused on communication and documentation of patient preferences for ICU teams, the strengthening of the role of the multidisciplinary team, and standardized comfort‐focused interventions during the last 48 h of life.

## 7 Conclusion

Interventions intended to modify the course of the disease exceeded those aimed at palliative care. Patients presented complications and such a severe critical condition prior to the last 48 h of life that no significant deterioration occurred that could clearly indicate the onset of terminality. Perceiving the final 48 h and the dying process becomes difficult when patients remain critically ill from admission or for a prolonged period before death.

Despite the severity of illness, high burden of comorbidities and frequency of complications, there was a low rate of formal identification of these needs, limited involvement of specialized palliative care teams, and under‐documentation of patients' and families' wishes regarding end‐of‐life decisions.

## Funding

The authors have nothing to report.

## Ethics Statement

This study was approved by the Research Ethics Committee (Approval No. 7453129).

## Consent

The data were obtained from secondary sources (electronic medical records), and no patients or healthcare professionals responsible for the records were identified.

## Conflicts of Interest

The authors declare that they have no known competing financial interests or personal relationships that could have appeared to influence the work reported in this paper.

## Data Availability

The data that support the findings of this study are available on request from the corresponding author. The data are not publicly available due to privacy or ethical restrictions.
